# Gender and Age Impacts on the Association Between Thyroid Function and Metabolic Syndrome in Chinese

**DOI:** 10.1097/MD.0000000000002193

**Published:** 2015-12-18

**Authors:** Zhaowei Meng, Ming Liu, Qing Zhang, Li Liu, Kun Song, Jian Tan, Qiang Jia, Guizhi Zhang, Renfei Wang, Yajing He, Xiaojun Ren, Mei Zhu, Qing He, Shen Wang, Xue Li, Tianpeng Hu, Na Liu, Arun Upadhyaya, Pingping Zhou, Jianping Zhang

**Affiliations:** From the Department of Nuclear Medicine (ZM, JT, QJ, GZ, RW, YH, SW, XL, TH, NL, AU, PZ, JZ), Department of Endocrinology and Metabolism (ML, XR, MZ, QH), and Department of Health Management, Tianjin Medical University General Hospital, Tianjin, P.R. China (QZ, LL, KS).

## Abstract

The relationship between thyroid dysfunction and metabolic syndrome (MS) is complex. We aimed to explore the impact of gender and age on their association in a large Chinese cohort.

This cross-sectional study enrolled 13,855 participants (8532 male, 5323 female), who self-reported as healthy without any known previous diseases. Clinical data including anthropometric measurements, thyroid function, and serum metabolic parameters were collected. The associations between thyroid function and MS of both genders were analyzed separately after dividing thyroid-stimulating hormone (TSH), free triiodothyronine (FT3), and age into subgroups. MS risks were calculated by binary logistic regression models.

Young males had significantly higher MS prevalence than females, yet after menopause, females had higher prevalence than males. Females had higher incidence of thyroid dysfunction than males. By using TSH quartiles as the categorical variables and the lowest quartile as reference, significantly increased MS risk was demonstrated in quartile 4 for males, yet quartiles 3 and 4 for females. By using FT3 quartiles as the categorical variables, significantly increased MS risk was demonstrated in quartile 2 to 4 for females only. By using age subgroups as the categorical variables, significantly increased MS risk was shown in both genders, with females (4.408–58.455) higher than males (2.588–4.943).

Gender and age had substantial influence on thyroid function and MS. Females with high TSH and high FT3 had higher MS risks than males. Aging was a risk for MS, especially for females. Urgent need is necessary to initiate interventional programs.

## INTRODUCTION

The metabolic syndrome (MS) is a cluster of interrelated metabolic abnormalities, which is characterized by central obesity, hyperglycemia, hypertriglyceridemia, decreased high-density lipoprotein-cholesterol (HDL), and elevated blood pressure (BP). People with MS have an increased risk of cardiovascular disease, type 2 diabetes mellitus, and all-cause mortality. MS was first defined in 1998,^1^ which was then recognized by the American Heart Association and National Heart, Lung and Blood Institute in 2001.^2^ In 2005, MS definition was updated by American Diabetes Association to meet fasting glucose (FG) standard and to tailor waist circumference (WC) cut-points to specific ethnicity.^3^ And in 2009, a consensus criterion was reached by a joint statement from the above organizations,^4^ which was most commonly referred to nowadays. MS requires 3 of the following 5 factors to make a diagnosis: increased WC, elevated triglycerides (TG), reduced HDL, elevated BP, and elevated FG. Reports from China show that a large proportion of Chinese are suffering from MS, which has become an important health concern in China.^5–8^ Rapid economic development with accelerating changes in urbanization, nutrition, lifestyle, socio-economic status, and reduced physical activity must play crucial roles in the dramatic escalation of MS in China.^5^ Sex and age are also key factors in the development of MS. Sex difference in MS prevalence has been noticed, but some reports showed higher prevalence in men,^7,8^ while others showed higher prevalence in women.^5,6^ Besides, aging has been demonstrated with increasing MS prevalence.^9,10^ Therefore, we consider more investigation is needed to clarify the gender disparity, and confirm the role of aging.

There is a female preponderance in thyroid disorders, and its prevalence increases with age. Thyroid hormones have pleiotropic effects on lipid and glucose metabolism, blood pressure, and energy expenditure. Thyroid dysfunction is a risk factor of cardiovascular disease.^11^ Recently, serum thyroid-stimulating hormone (TSH) is also found to be associated with adverse changes of lipid metabolism as well.^12,13^ The relationship between mild thyroid dysfunction and MS traits has become a hot topic of discussion recently, because both could increase morbidity and mortality. Patients with hypothyroidism^14^ and subclinical hypothyroidism^15^ were identified to have increased risks of MS. Even in euthyroid subjects, high normal TSH levels (>2.5 μIU/mL) were significantly associated with an increased prevalence of MS.^16,17^ Ruhla et al^16^ indicated that a TSH below 2.5 μIU/mL was associated with a favorable metabolic profile. Oh et al^17^ advocated that if healthy women had a TSH higher than 2.5 μIU/mL, assessment of MS should be done. However, there are reports with discordant results, which could not demonstrate such associations between TSH and MS.^18,19^ This inconsistency also warrants further analytical investigation on a bigger population.

The objective of our cross-sectional study was to investigate correlations between thyroid dysfunction and MS with emphasized focuses on differences generated from gender and age in a representative sample of Tianjin municipality population.

## METHODS

### Design

This cross-sectional, community-based health-check investigation was conducted in Tianjin Medical University General Hospital, under collaboration from the departments of Health Management, Endocrinology & Metabolism, and Nuclear Medicine, as reported before.^20–22^ All participants were required to complete a questionnaire and provide a blood sample. They were self-reported as healthy. In order to avoid the influence of confounding factors, the following criteria were used for exclusion: subjects with disease history of thyroid, liver, kidney, gastro-intestine, or oncology; subjects with any diseases or taking any medicine that might affect thyroid or metabolism (eg, antithyroid drugs, thyroid hormone, amiodarone, iodine, estrogen, androgen, statins, steroid hormones, etc.); and pregnancy. During the period from September 2011 to March 2014, a total of 13,855 eligible subjects (8532 male, 5323 female) had adequate data for analysis. Written consents were obtained, and the institutional review board and ethic committee of Tianjin Medical University General Hospital approved this study.

### Measurements

Anthropometric measurements and fasting blood tests of the participants were performed during their visits to our institution. Body height and body weight (BW) were measured in centimeters and kilograms. Body mass index (BMI) was calculated by dividing BW (kg) by the square of body height (m^2^). Fasting blood samples were obtained between 7 am and 10 am. TSH, free triiodothyronine (FT3), and free thyroxine (FT4) were analyzed on a fully automated ADVIA Centaur analyzer (Siemens Healthcare Diagnostics, Erlangen, Germany) by chemiluminescent reaction principle. Total cholesterol (TC), TG, low-density lipoprotein-cholesterol (LDL), HDL, alanine aminotransferase (ALT), total bilirubin, blood urea nitrogen (BUN), uric acid (UA), creatinine (Cr), and FG were determined enzymatically by an auto-analyzer (Hitachi Model 7600 analyzer, Hitachi, Tokyo, Japan).

The laboratory reference ranges for parameters were as follows: TSH 0.3 to 5.0 μIU/mL; FT3 3.5 to 6.5 pmol/L; FT4 11.5 to 23.5 pmol/L; TC 3.59 to 5.18 mmol/L; TG 0.57 to 1.70 mmol/L; LDL 1.33 to 3.37 mmol/L; HDL 0.8 to 2.2 mmol/L; ALT 5 to 40 U/L; total bilirubin 3.4 to 20 μmol/L; BUN 1.7 to 8.3 mmol/L; UA 140 to 414 μmol/L; Cr 44 to 115 μmol/L; and FG 3.6 to 5.8 mmol/L.

## DEFINITION

MS was diagnosed by the consensus criterion, which required at least 3 of the followings^3,4^: WC ≥90 cm in men, ≥80 cm in women; TG ≥ 1.70 mmol/L; HDL <1.03 mmol/L in men, <1.29 mmol/L in women; systolic blood pressure (SBP) ≥130 mmHg or diastolic blood pressure (DBP) ≥85 mmHg; and FG ≥ 5.6 mmol/L.

Thyroid function subgroups were determined in 2 methods. In the first method, hyperthyroidism was defined as TSH ≤ 0.3 μIU/mL, hypothyroidism as TSH > 5.0 μIU/mL. Then hypothyroidism was further divided into mild hypothyroidism subgroup (5.0 μIU/mL < TSH ≤ 10.0 μIU/mL) and overt hypothyroidism subgroup (TSH > 10.0 μIU/mL). Because upper reference of 2.5 μIU/mL was recommended by guidelines,^23,24^ euthyroidism was further divided into perfect function subgroup (0.3 μIU/mL < TSH ≤ 2.5 μIU/mL) and normal function subgroup (2.5 μIU/mL < TSH ≤ 5.0 μIU/mL). In the second method, TSH, FT3, and FT4 were divided based on quartiles of the measurements. Age subgroups 1 to 6 were defined according to the followings, respectively: age ≤ 25 years, 25 years < age ≤ 35 years, 35 years < age ≤ 45 years, 45 years < age ≤ 55 years, 55 years < age ≤ 65 years, age > 65 years.

### Statistical Analysis

All data were presented as mean ± standard deviation. Differences of indices between groups or subgroups were analyzed by independent sample's *t*-test. Chi-square test was used to compare intergroup prevalence differences. Pearson bivariate correlation was made among variables. Odds ratios (ORs) for MS with 95% confidence intervals were calculated by binary logistic regression models. Statistical Package for Social Sciences (SPSS version 17.0, Chicago, IL) was used to conduct statistics and significance was defined as *P* < 0.05.

## RESULTS

### Characteristics of the Participants in Different Genders

There were significant differences in all parameters with respect to opposite gender (Table 1). Males were younger than females, yet BMI, MC, SBP, and DBP in males was higher than in females. TSH was lower in males than in females, while FT3 and FT4 were higher in males than in females. TC and HDL were lower in males than in females, yet TG and LDL were higher in males than in females. Besides, indices representing hepatic and renal functions were higher in males than in females.

**TABLE 1:**
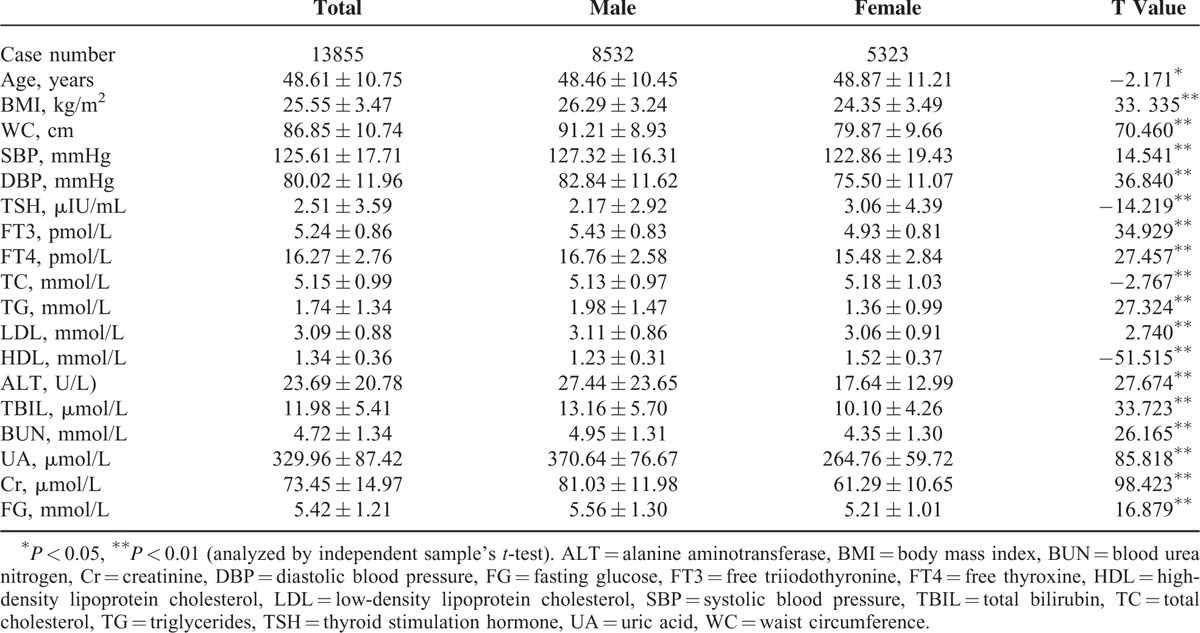
Population Characteristics Based on Different Genders

### Prevalence of MS in Different Genders

**FIGURE 1:**
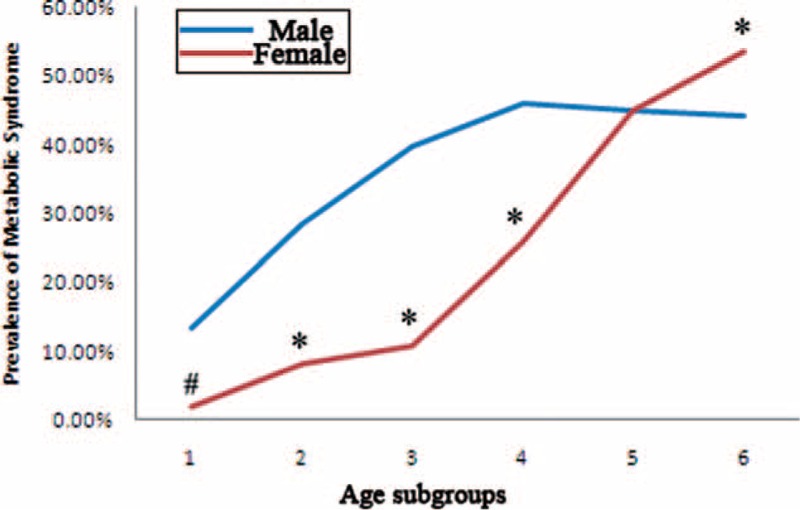
Prevalence of MS in different age subgroups. Age subgroups 1 to 6 referred to the followings: age < 25 years, 25 years ≤ age < 35 years, 35 years ≤ age < 45 years, 45 years ≤ age < 55 years, 55 years ≤ age < 65 years, age ≥ 65 years. MS = metabolic syndrome. #, difference of prevalence between gender was significant at 0.05; ∗, difference of prevalence between gender was significant at 0.01.

Overall prevalence of MS was 35.55% (4926/13,855) in our population. Males (41.95%, 3580/8532 cases) had significantly higher MS prevalence than females (25.29%, 1346/5323 cases), with a Chi-square value of 195.341 (*P* < 0.01). Age cast significant differences on the prevalence of MS, rendering a crisscross pattern (Fig. 1). The prevalence of MS showed an increasing tendency in males from the youngest age to the age range of 45 to 55 years, and then stayed roughly in the plateau level till the highest age subgroup (Chi-square value = 101.954, *P* < 0.01). In females, MS prevalence tendency increased from the youngest to the eldest, while the significantly sharp increase of MS prevalence started from the middle age to the eldest (Chi-square value = 676.323, *P* < 0.01). Males, with the age of younger than 45 years, had significantly higher MS prevalence than females (*P* < 0.01). However, after menopause (older than 65 years), females had significantly higher MS prevalence than males (*P* < 0.01).

**FIGURE 2:**
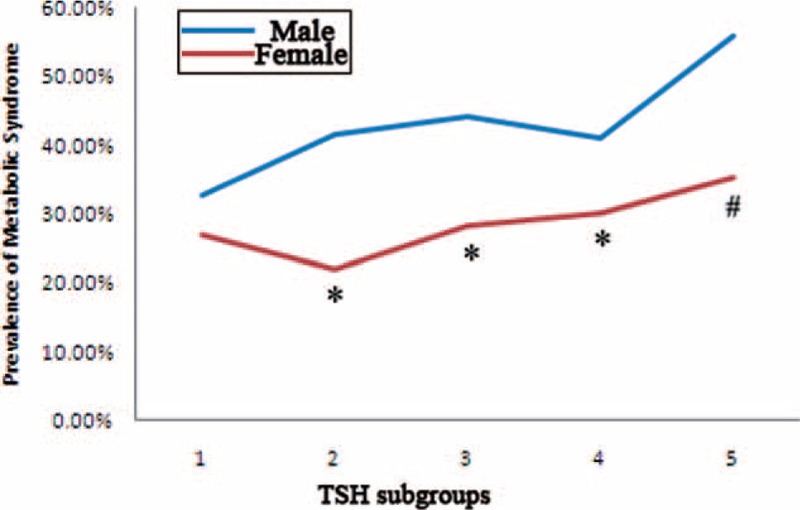
Prevalence of MS in different TSH subgroups. TSH subgroups 1 to 5 referred to the followings: TSH ≤ 0.3 μIU/mL, 0.3 μIU/mL < TSH ≤ 2.5 μIU/mL, 2.5 μIU/mL < TSH ≤ 5.0 μIU/mL, 5.0 μIU/mL < TSH ≤ 10.0 μIU/mL, TSH > 10.0 μIU/mL. MS = metabolic syndrome, TSH = thyroid stimulating hormone. #, difference of prevalence between gender was significant at 0.05; ∗, difference of prevalence between gender was significant at 0.01.

MS prevalence according to various thyroid functional status demonstrated different patterns (Fig. 2). First, MS prevalence was significantly higher in males than in females in all thyroid functional status (*P* < 0.01), except for hyperthyroidism when TSH < 0.3 μIU/mL. Second, MS prevalence displayed a rising trend along with the increase of TSH levels, especially for women (for males, Chi-square value = 9.335, *P* = 0.053; for females, Chi-square value = 34.876, *P* < 0.01).

### Incidence of Thyroid Dysfunction in Different Genders

Our population demonstrated an incidence of thyroid dysfunction of 6.80% (942/13,855), with hypothyroidism of 5.90% (818/13,855) and hyperthyroidism of 0.89% (124/13,855), the former significantly higher than the latter (Chi-square value = 494.771, *P* < 0.01). Females had significantly higher overall incidence of hypothyroidism and hyperthyroidism than males (Table 2). When we analyzed the detailed incidences divided by age, most age subgroups demonstrated the same pattern of differences between the opposite gender, except for the people of the youngest age. However, the fact that there were only a very small number of participants in the age subgroup of ≤25 years, the incidences calculated here might not be representative. Another important finding was that there existed a significant tendency of increasing hypothyroidism incidence with aging (except for the people of the youngest age) for both gender. Chi-square test showed significances in males (Chi-square value = 85.487, *P* < 0.01) and in females (Chi-square value = 60.820, *P* < 0.01).

**TABLE 2:**
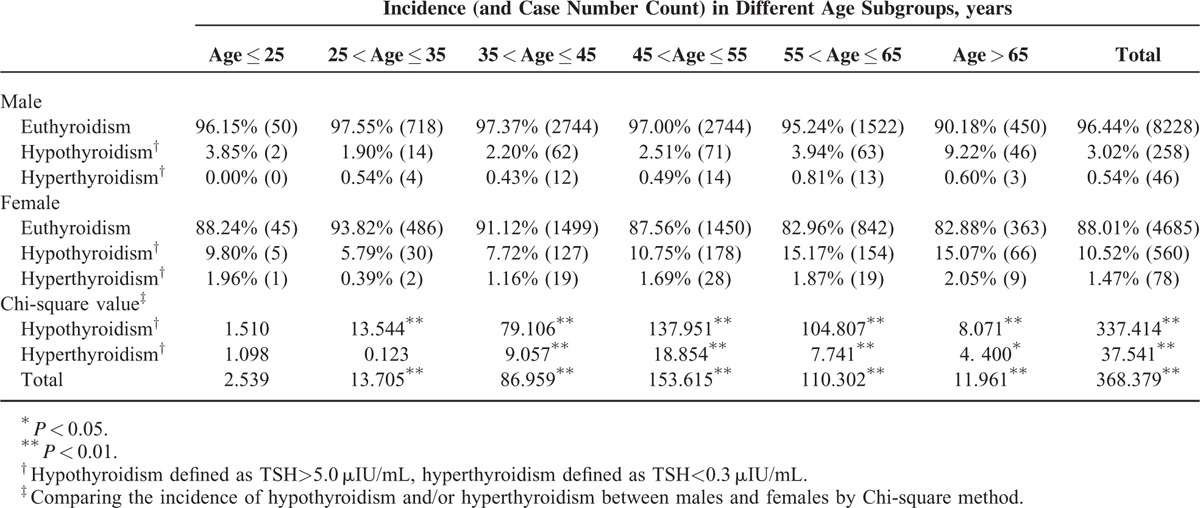
Incidence of Hypothyroidism and Hyperthyroidism on Different Genders

### Correlations of Key Variables in Different Genders

Age was indicated to have correlations with nearly all of the variables, and correlation coefficients were significantly higher (Table 3). Age and BMI showed positive correlation in females yet negative correlation in males. In both genders, age and TSH showed positive correlation, while age and FT3 showed negative correlation. Age showed positive correlation with WC, SBP, DBP, TC, LDL, BUN, Cr, and FG in both genders, yet negative correlation with TG in males and negative correlation with HDL in females.

**TABLE 3:**
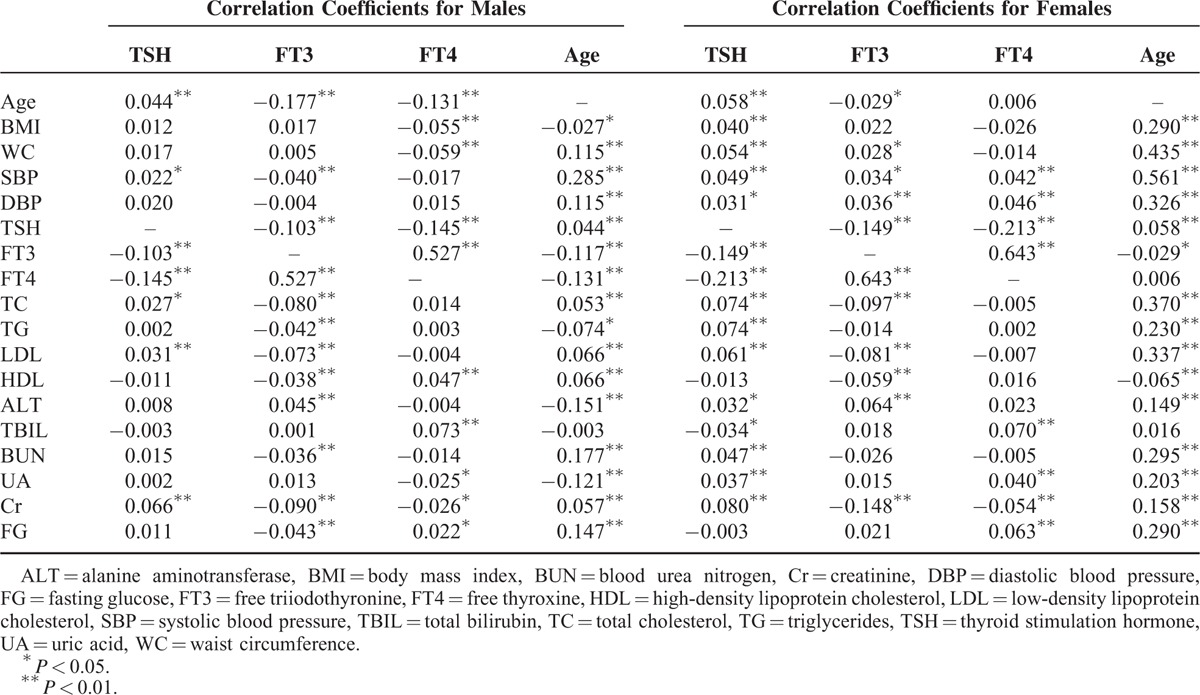
Pearson Bivariate Correlations Among Key Variables Based on Different Genders

TSH demonstrated significant negative relationships with FT3 and FT4 for both genders. In males, positive relationships were demonstrated for age, SBP, TC, LDL, and Cr. In females, positive relationships were demonstrated for age, BMI, WC, SBP, DBP, TC, TG, LDL, ALT, BUN, UA, and Cr.

### Risks of Developing MS in Different Genders

Five binary logistic regression models were utilized to calculate the risks of developing MS (Table 4). The 1st model designated thyroid functional states as the categorical variables, and the subgroup of TSH < 0.3 μIU/mL was determined as reference. Age, FT3, and FT4 were included as covariates. This model did not reveal any significant risks of MS in either sex. The 2nd model designated TSH quartiles as the categorical variables, and the lowest quartile was determined as reference. Significantly increased risk was demonstrated in quartile 4 for males, while in quartiles 3 and 4 for females. The 3rd model designated FT3 quartiles as the categorical variables, and the lowest quartile was determined as reference. Age, TSH, and FT4 were included as covariates. Significantly increased risk was demonstrated in quartile 2 to 4 for females, yet no risk was displayed in males. The 4th model designated FT4 quartiles as the categorical variables, and the lowest quartile was determined as reference. Age, TSH, and FT3 were included as covariates. This model did not indicate any significant risks of MS in either sex. The 5th model designated age subgroups as the categorical variables, and the subgroup of age ≤25 years was determined as reference. TSH, FT3, and FT4 were included as covariates. Significantly increased risks were shown in both genders, with ORs in females (4.408–58.455) higher than ORs in males (2.588–4.943).

**TABLE 4:**
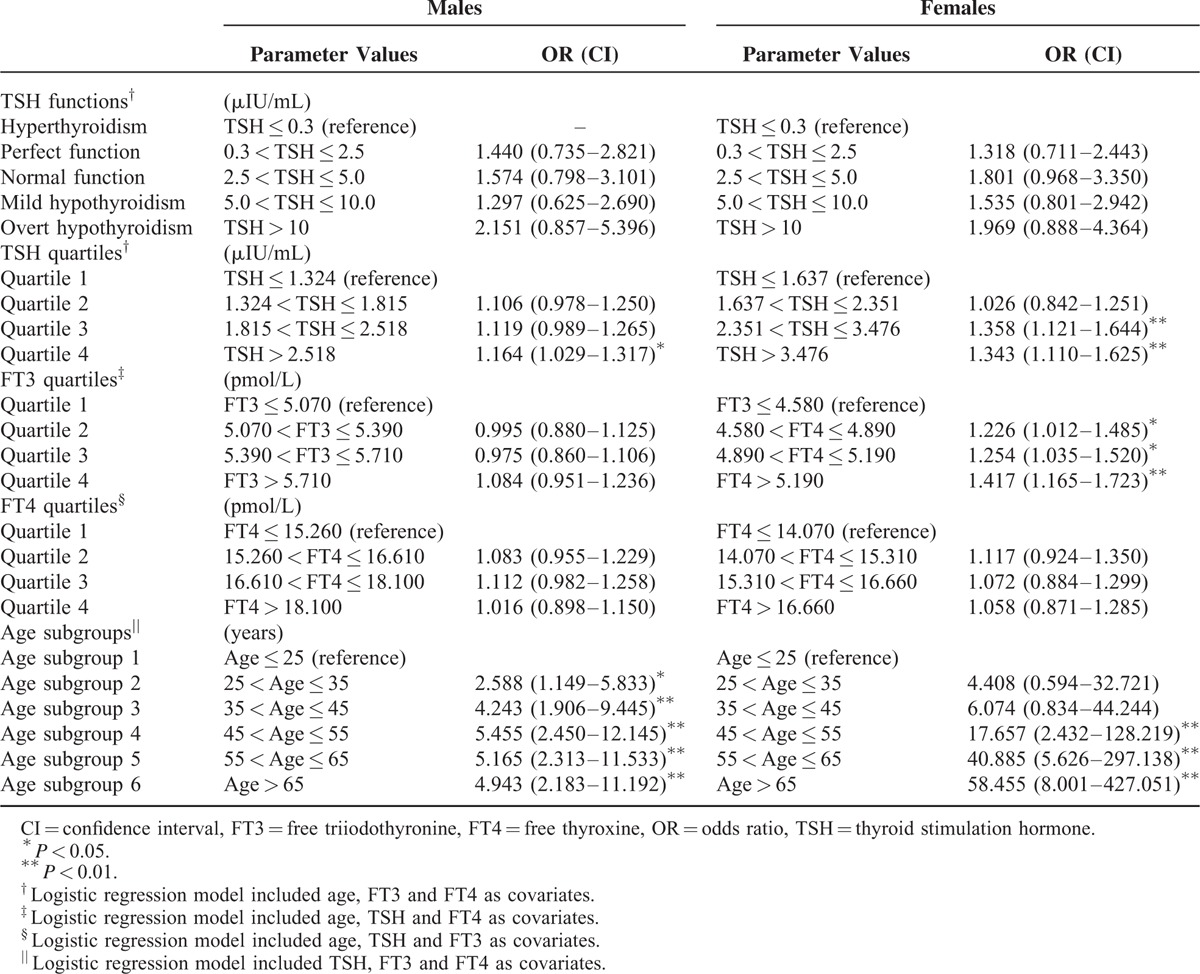
The Risks of Developing MS in Different Genders

## DISCUSSION

During recent decades, nutritional, demographic, epidemiological, and socioeconomic transitions have been occurring in China. Lifestyle of large proportions of the Chinese population has been drastically changed to an excessive energy-dense diet intaking and sedentary occupational environment pattern. In parallel with the lifestyle changes, dysmetabolic phenotypes, such as hyperglycemia, obesity, and hypertension, are rising to epidemic levels.^5–8^ WHO estimates that, globally, there are more than 1 billion adults overweight and 300 million obese people.^25^ MS defines a constellation of such metabolic risk factors, it is estimated at least 1 in 3 adults in USA meet the MS criteria.^26^ In developing countries, the prevalence of MS also increases. Misra and Khurana^25^ compiled the data and reported MS prevalence in developing countries in 2008, for example, sub-Saharan Africa and Middle East countries (33.5%), India (28.8%), Turkey (33.4%), Iran (33.7%), Venezuela (31.2%), and Brazil (25.4%). MS is associated with an increased risk of diabetes, and a number of cardiovascular events, such as myocardial infarction, stroke, and heart failure.^27^ The cost of metabolic abnormality is also astronomical. In mainland China, the total medical costs attributable to overweight and obesity alone were estimated at 21.1 billion Yuans in 2003, accounting for 3.7% of national total medical costs.^28^ This figure was also referred in the review by Huang et al,^29^ who pointed out that obesity and tobacco were 2 leading causes of several noncommunicable diseases, and obesity was attributable to 363,000 mortality and 12.3 million disability-adjusted life years. Alarmingly, in 2010, overweight and obesity were calculated to be responsible for 42.9% of the medical and nonmedical yearly costs of the major noncommunicable diseases in China, with a substantial amount of 90.8 billion Yuans, which accounted for 4.5% of the national health expenditure.^30^

Metabolism has a sex dimorphism, which results in different cardiovascular disease risks for men and women. In particular, the reduced risk of cardiovascular disease in women compared to men before menopause has been speculated to be related to a reduced abdominal fat accumulation in women.^31^ Yet, after menopause, with the decline of circulating estrogens, the concentrations of lipoproteins, as well as body fat distribution in females will shift toward a central or android pattern more similar to males.^32^ The current investigation showed a crisscross pattern of MS prevalence. Young men had significantly higher overall MS prevalence than young women, yet after menopause, MS prevalence in old women surpassed old men. Previous reports also displayed the same results.^7,8^ During menopause, alterations in sex hormones are obvious. Decline in ovarian function ensues a reduction in estrogen production, yet androgen synthesis by the adrenal cortex is less affected. This might be the crucial reason leading to MS risk changes. Menopause is associated with an increase in all single components of the MS (ie, with the development of android obesity, hypertension, degradation in the lipid profile, and hyperinsulinemia).^33^ The overall predictive power and prognostic significance of MS for adverse events of cardiovascular diseases and all-cause mortality were higher in women than in men.^34^

Males and females have different propensities for thyroid dysfunction, which are not the same as metabolic abnormality. Various forms of thyroid dysfunction and Hashimoto thyroiditis are more common in women, and the overall incidence increases with age in both sexes (TSH distributional shift with age), especially in women.^35–37^ Our results confirmed that females had significantly higher incidence of hypothyroidism and hyperthyroidism than males. In a screening population like in our study, mild thyroid dysfunction is discovered very frequently, since most of such patients are asymptomatic, so they do not seek for medical help on their thyroid problems beforehand.^36,37^ Additionally, current annual health check program in China is not usually comprehensive enough to cover thyroid assessment for most people. However, not only overt thyroid dysfunction, but also mild thyroid dysfunction shows increased risk of coronary heart disease and mortality,^36–38^ which should not be overlooked at all.

Thyroid hormones play important roles in metabolism, and cardiovascular system is also very sensitive to thyroid function. The mechanistic explanations for the relationship between thyroid hormones and MS have not been fully elucidated, although the effects are believed to be mediated at both genomic and nongenomic levels.^39^ There are several possible postulations put forward as the followings. Thyroid hormones can play a role in the development of insulin resistance,^40,41^ which is considered as an essential causative factor and basis for MS.^1,2,42^ Another obvious phenomenon is all MS components are intrinsically associated with thyroid, which eventually leads to MS as a final result. For instance, hypothyroidism can increase body weight, and TSH is identified to be positively related with BMI and obesity, even within the normal range.^43^ Increasing TSH in overt and mild hypothyroidism is also found to be associated with unfavorable lipid concentrations (like hypercholesterolemia and decreased HDL levels),^11,15^ as well as blood pressure.^44^ Thyroid function also has an effect on the activity of cholesteryl ester transfer protein and hepatic lipase,^45^ which alters HDL metabolism. As a result, T3 is negatively associated with HDL.^46^

Aging is defined as a series of morphological and functional changes taking place overtime, which denotes deterioration of the biological functions after an organism has attained its maximal reproductive potential. The most important gender differences in aging are related to the reproductive organs. Women experience a rapid decline in sex hormones during the cessation of menses, while men experience a slow and continuous decline.^10^ Age is also an important factor influencing the association between MS and thyroid function. In fact, aging of the population was regarded by Lao et al^5^ as one of the major factors driving dramatic MS escalation. Bensenor et al^47^ demonstrated that aging promotes a natural decrease in the pituitary TSH secretion and deiodination of T4, while increases the occurrence of antithyroglobulin and antithyroperoxidase antibodies. During aging, reproductive and nonreproductive actions of sex steroid hormones decrease significantly. Gender differences in prevalence, time of onset and severity of MS, and its cardiovascular consequences are results of the different rate of decrease of sexual hormones in males and females.^10^ We showed that aging resulted in increased prevalence MS and thyroid dysfunction (Fig. 1 and Table 2). Our investigation was in line with the previous reports.^5,36^ We also demonstrated that the increase rate of MS in women from before menopause to after menopause was very dramatic, rendering a crisscross pattern (Fig. 1), which was in confirmative with our previous study^22^ and others.^8^ Binary logistic regression models reveled that aging was much more effective than thyroid function parameters (TSH or FT3) to engender an enhanced risk of MS (Table 4).

One of our findings was that higher FT3 was a risk factor for MS, which appeared contradictory to another of our findings that higher TSH was a risk factor for MS. In fact, this paradoxical phenomenon has been noticed by several previous investigations.^46,48,49^ Positive associations between FT3 and metabolic status (eg, BMI) were reported, this is in contrast to FT4, which had negative relation with metabolic traits. And higher FT3 level was reported to be associated with various markers of unfavorable metabolic profile and cardiovascular risk.^46,48,49^ Roef et al^46^ showed that in healthy euthyroid middle-aged men and women, higher FT3 levels, lower FT4 levels, and thus a higher FT3-to-FT4 ratio were consistently associated with various markers of unfavorable metabolic profile and cardiovascular risk. Ortega et al^49^ demonstrated that FT3, not FT4, concentrations were associated with direct and indirect measurements of insulin secretion, independent of insulin sensitivity, and glucose concentrations in euthyroid individuals. This study indicated that T3 concentrations might play a role in the regulation of insulin secretion. The main mechanism of this phenomenon has been considered as that changes in deiodinase 1 and/or 2 catalytic properties in subjects with higher fat mass and unfavorable metabolic profile lead to higher conversion of T4 to T3.^50^ Indeed, Ortega et al^50^ demonstrated that activity of deiodinases 1 was increased in omental and subcutaneous fat of obese subjects when compared with nonobese subjects and correlated well with BMI. Increased levels of deiodinases 1 in adipose tissue of obese subjects might reflect the stimulation of this enzyme by locally formed leptin. And, in turn, enhanced production of T3 by deiodinases 1 in fat could be involved in the modulation of adipose tissue metabolism by leptin. As for other possible mechanisms, Roef et al^46^ postulated that an adverse metabolic profile could theoretically lead to an altered thyroid hormone secretion by the thyroid gland itself, with a relatively higher T3-secretion. Besides, since both higher TSH levels as well as a higher FT3-to-FT4 ratio could indicate a lower iodine pool, and since TSH was also positively related to the FT3-to-FT4 ratio, another mechanism underlying the higher FT3-to-FT4 ratio (and the higher TSH levels) in subjects with a less favorable metabolic profile could be a lower iodine intake in the diet or a decreased absorption.

The net effect of gender and age on the association between thyroid dysfunction and MS is the most important objective of our analysis. We found that women have more increased risks of developing MS than men, under the circumstances of high TSH, high FT3, and advanced age (Table 4). We consider the reason of these results could be that estrogen's protective effects on MS are overshadowed and overwhelmed by the female inclination of thyroid dysfunction. When women age, postmenopausal phenomenon will have compromised estrogen milieu, yet enhanced thyroid abnormality. Under such circumstances, increased changes of TSH and FT3 can herald MS risks. MS and thyroid dysfunction are 2 common endocrine disorders with a substantial overlap, and both are associated with significant morbidity and mortality. Both diseases are becoming major public health problems in China, which underscore the urgent need to develop comprehensive strategies aimed at the prevention and treatment of the diseases to reduce increased socioeconomic, medical and societal burdens, and ramifications in China. Urgent public health actions are needed to control this challenge in China.

Limitations of our study deserve additional comments. First, this was a cross-sectional study, so a cause and effect relationship cannot be discerned. Further prospective studies should be carried out to answer the causality question. Second, we did not obtain data of thyroid antibodies, which might influence thyroid hormone. So, we could not comment on the putative impact of thyroid autoimmunity. Third, we did not measure sex hormones in our population because of the budget shortage. Finally, we checked TSH only once, we did not double check TSH measurement in the study.

In conclusion, we found that serum TSH and FT3 levels were positively associated with MS. And females had higher risks than males. Moreover, aging is an important risk factor for MS. Our results suggest the necessity of monitoring TSH and FT3 in the population for MS risk assessment, especially elder women.
